# Coverage Path Planning Using Reinforcement Learning-Based TSP for hTetran—A Polyabolo-Inspired Self-Reconfigurable Tiling Robot

**DOI:** 10.3390/s21082577

**Published:** 2021-04-07

**Authors:** Anh Vu Le, Prabakaran Veerajagadheswar, Phone Thiha Kyaw, Mohan Rajesh Elara, Nguyen Huu Khanh Nhan

**Affiliations:** 1 ROAR Lab, Engineering Product Development, Singapore University of Technology and Design, Singapore 487372, Singapore; leanhvu@tdtu.edu.vn (A.V.L.); prabakaran@sutd.edu.sg (P.V.); rajeshelara@sutd.edu.sg (M.R.E.); 2 Optoelectronics Research Group, Faculty of Electrical and Electronics Engineering, Ton Duc Thang University, Ho Chi Minh City 700000, Vietnam; 3 Department of Mechatronic Engineering, Yangon Technological University, Insein 11101, Myanmar; mlsdphonethk@gmail.com

**Keywords:** reconfigurable system, tiling robotic, reinforcement learning TSP, complete path planning, energy-aware reward function

## Abstract

One of the critical challenges in deploying the cleaning robots is the completion of covering the entire area. Current tiling robots for area coverage have fixed forms and are limited to cleaning only certain areas. The reconfigurable system is the creative answer to such an optimal coverage problem. The tiling robot’s goal enables the complete coverage of the entire area by reconfiguring to different shapes according to the area’s needs. In the particular sequencing of navigation, it is essential to have a structure that allows the robot to extend the coverage range while saving energy usage during navigation. This implies that the robot is able to cover larger areas entirely with the least required actions. This paper presents a complete path planning (CPP) for hTetran, a polyabolo tiled robot, based on a TSP-based reinforcement learning optimization. This structure simultaneously produces robot shapes and sequential trajectories whilst maximizing the reward of the trained reinforcement learning (RL) model within the predefined polyabolo-based tileset. To this end, a reinforcement learning-based travel sales problem (TSP) with proximal policy optimization (PPO) algorithm was trained using the complementary learning computation of the TSP sequencing. The reconstructive results of the proposed RL-TSP-based CPP for hTetran were compared in terms of energy and time spent with the conventional tiled hypothetical models that incorporate TSP solved through an evolutionary based ant colony optimization (ACO) approach. The CPP demonstrates an ability to generate an ideal Pareto optima trajectory that enhances the robot’s navigation inside the real environment with the least energy and time spent in the company of conventional techniques.

## 1. Introduction

Cleaning by covering the workspace has been fundamental for a friendly ecosystem but a tedious workload for humans. Over the long haul, automated cleaning devices are gradually being invented. Designing autonomous mobile robots is the fundamental concept of complex intelligent navigation systems. [1]. The author in [2] addresses all the pertinent topics of the electronic hardware and software of the mobile robot design, emphasizing the more complex problems. Recently, with the development of advanced robotic technologies such as precision mechanics, artificial intelligence, a significant number of cleaning systems have routinely implemented cleaning tasks in indoor and public spaces. Specifically, there are numerous floor cleaning robots operating in indoor environments in the market, but they are all in the fixed morphology of circle, space, and oval, and struggle to cover the complex indoor environments. Moreover, most cleaning systems in the market provide manual or semi-auto modes that only work effectively under constrained environments. It has been seen that there are many degrees for robotizing cleaning undertakings in-home establishments. The business of cleaning gadgets for homes has been on the rapid ascent in recent years. Even though they are financially profitable, their immobilization keeps them from accomplishing the most significant cleaning limitations.

Reconfigurable robot platforms can be categorized into three major classes [3]: intra-reconfigurable; inter-reconfigurable; and nested reconfigurable robots. The development of reconfigurability in robotics has received increasing attention, and platforms with a wide variety of reconfigurable mechanics have been deployed [4].

The reconfigurable tiling robot’s fundamental objective is to augment the working areas’ constraints that limit the fixed form robots. Their capacity to change morphology causes them to accomplish their objectives of covering the workspace completely. There are various reconfigurable tiled robots created by specialists from the academic to industrial environment to perform the cleaning of different environments [5]. The robots referenced herein can change into various shapes. The novel reconfigurable tiling robot [6] can change into seven shapes, and the other robots [7,8] can change into three shapes. In the current paper, hTettran, a polyabolo-inspired self-reconfigurable tilling robot, was utilized to validate our reinforcement learning (RL) path planning a proposed algorithm. Reconfigurability gives the robot the benefit of moving around deterrents and can likewise access the spaces that are difficult to clean.

The complete path planning (CPP) approaches were executed on many static form robots, one of which was referenced in [9]. The authors designed a total cover course arranging and directing a technique for versatile mechanical robots to clean the enormous zones. The authors in [10] introduced a technique for novel reconfigurable robots deploying in the de-mining, cleaning, and painting applications. They actualized the cell decomposition to simplify a specific known territory into various cells based on the complexities of sub-regions. In the paper [11], the creators introduced an altered form of the A* path planning techniques in which the proposed rendition makes appropriate robot footprints expecting the e-morphology generation of the tiling robot focusing on covering the narrow constraint spaces [6]. A few scientists have been attempting to conduct various area coverage applications with adaptive CPP by automated vehicles that can be utilized in different fields and terrain [12,13].

RL has been connected in different areas to induce the ideal arrangement consequently in the ship hull surface cleaning works [14]. Changxi et al. [15] has proposed utilizing RL as CPP to indirectly self-investigate the workspace in uneven terrain. Kenzo et al. [16] utilized the RL model with design preknowledge-based reward function to plan bipedal robots’ movement in football arrangement. Farad et al. [17] has made a way to yield the optimal capability under the constraint conditions of coverage of the complex environment through the RL. The idea of utilizing Q-learning with the obstacle aware to generate the shortest track from the source to destination in a grid-based divided sub-region was been proposed in Aleksandr et al. [18], Amit et al. [19] and Soong et al. [20]. David et al. amplifies this strategy to different robot specialists [21]. Yuan et al. [22] utilized the RNN gated recurrent unit (GRU) framework to plan an ideal way from the source to the destination straightforwardly.

A common part of the CPP approach was the simplification of specific territories/maps into cells. Numerous traditional strategies have been proposed for map simplification purposes by decomposing. The fundamental system divides the complicated map into more modest locales called sub-zones or plots [23]. The conventional technique is to partition a given territory by basic shapes like isolated triangles, trapezoid, grid-cell utilizing Morse capacities, 3D information [24,25]. Analysts have applied various methodologies to decode the maps, as referenced previously. The recent paper utilizes network-based deterioration introduced by the author in [26]. Numerous techniques allow us to partition the workspace utilizing various learning-based methodologies, for example, tree scaling, energy acknowledgement calculations [27,28], AI-based deep learning [29] and grid-cell-based guide deterioration [30]. These approaches are lightweight in computational intricacy to make a decomposition map for effective CPP.

The overall proposed technique of the CPP for the proposed tiling robot platform called hTetrran consists of these steps: Initially, a tiled set is created depending on the available shapes of the robot to fit in the free space cells and constraints area of workspaces. In the case of the hTetran platform robot, this tileset is made depending on the polyabolo polyomino hypothesis with various theories along with their confirmation of complete coverage of the given workspace. After generating the tileset, the proposed hTetrran platform can navigate to different workspace locations while reconfiguring the defined shape to avoid obstacles. Therefore, calculations assuming the appropriate robot shape can ensure that each free space inside the defined workspace is covered by a proposed robot footprint. The progress here is that the usual tiled hypothetical calculation produces non-ideal puzzles. This means that the robot needs to accept the given forms roughly. For precise rendering, there may be examples in which the robot can clean a particular area by none reconfiguring is required at all. The tiled hypothesis could then suggest the robot to perform multiple configurations at each point repeatedly. This type of organization affects the robot’s energy use. The navigation sequence modelled as an NP problem of the travel salesman problem will link the generated tiles together in an appropriate direction. This can be done through calculations arranged conventionally, such as zigzag, spiral, and random search. However, their exhibit depends entirely on the workspace conditions and takes a considerable execution time. The motivation of optimal navigation sequence minimizes the required actions, hence reducing the energy usage and operation time. Another appropriate technique is to use evolutionary optimization, for example, ant colony optimization (ACO) [31], to derive the optimal solution for the defined travel sales problem (TSP) in an appropriate amount of execution time. However, the technique cannot be extended to a larger and complex environment. This technical rationalization also requires a lot of extensive computational costs, and the results could be problematic if, for this situation, at least the iteration optimal progress gets stuck at the local minima. The profile shows the possibility of finding a way for the robot in complex conditions by memorizing various deep learning references in predefined workspaces.

The current paper proposes a well-trained depth reinforcement learning model that addresses TSP sequencing optimization to cover the entire area using a polyabolo-inspired self-reconfigurable robot. The RL network’s reward function is designed to reflect the robot’s actual operation with the decomposed polyabolo workspace by the proposed tiling theory. The framework aimed to ideally realize a lower navigation cost linking the predefined polyabolo tileset locations compared to conventional evolutionary-based methods. The present paper is organized as follows: Section 2 describes the hTetran platform. Section 3 is the RL-based CPP; then Section 4 is the experimental results in both the simulation and real environments. The last section, Section 5, is the conclusion and future works.

## 2. The hTetran Platform Description

### 2.1. The hTetran System Architecture

**Figure 1:**
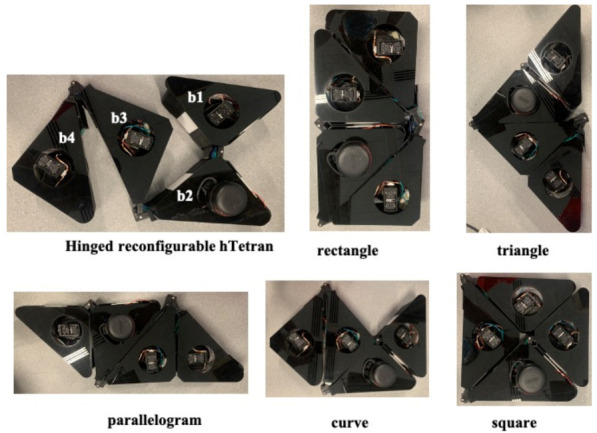
hTetran platform with shapeshifting to five morphologies.

The presented robot was developed using the principle of linked polyabolo-based blocks. The robot consisted of four isosceles right-angle triangular blocks connected with active hinges. We chose the right angle isosceles poly-form as our robot structure to achieve maximum area coverage by changing its defined morphologies among rectangle; triangle; parallelogram; curve; and square as shown in Figure 1.

**Figure 2:**
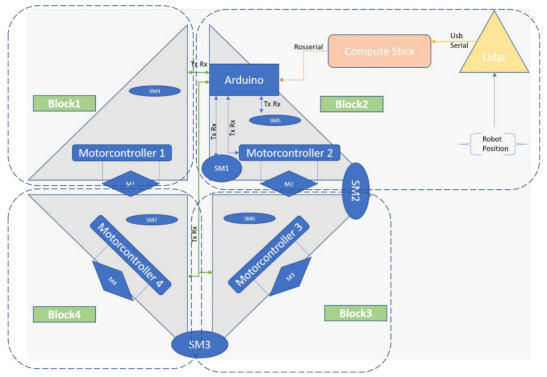
Electronic parts of hTetran platform.

The robotic device was categorized into several subsystems such as the locomotion, reconfigurable mechanism; structural design; and electronic circuits. This subsystem acts as an essential component that combines achieving environmental adaptation and obstacle detection. The structural dimension of each isosceles triangular block was developed with a dimension of 210 mm in adjacent and 294 mm in hypotenuse. The block’s vertices were positioned as chambers to skip the edge collision between the blocks during reconfiguration. The robot’s walls and base were constructed with an acrylic sheet of 2 mm thickness. The robot is equipped with a set of Herkulex motors and Pololu dc motors in each locomotion module. The Herkulex motor acted as a steering motor, and the dc motor drives the robots as in Figure 2. With such an arrangement, the robot could achieve holonomic locomotion as a soc. Each locomotion motor set was powered with 7.4 VDC battery. Concerning the reconfiguration, we again equipped two Herkulex servo motors housed in block 1 and block 2.

**Figure 3:**
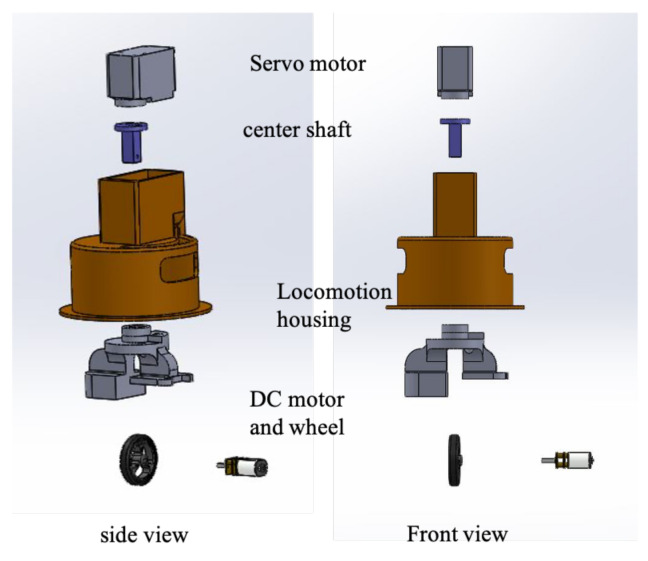
Locomotion unit of hTetran to archive holonomic movement.

The Herkulex motor could rotate with an angle of range of 320 degrees with a stall torque of 24 kgcm. Herkulex SM1 had a rotational angle limit of 180 degrees, housed between blocks 1 and 2. Similarly, the other Herkulex SM2 and SM3 had the rotational angle limit of 270, which was housed between blocks 2 and 3, and blocks 3 and 4, respectively. The hinged motors also acted as a lock for the robot to maintain the shape through the operation. We equipped most of our electronic components in block 2 since it acts as an anchor point and does not involve any reconfiguration process, as shown in Figure 3. The first principal component is the motor driver, which controls the Pololu motor housed in each block. We attached an Arduino mega controller in block 2, which acts as a low-level controller of the robot. The communication between the motor driver and the Arduino happens through serial communication pin 1. Other than the motor driver, we had another serial communication for Herkulex servo motors. The second serial communication was connected in pin 2. Other than serial communication, we used an I2C communication from Arduino to communicate with the IMU whose power was drawn from the Arduino board. For the power source, we placed a 12 VDC battery in block 2, which is connected in parallel to Arduino, the motor driver, and Herkulex motors. When it comes to higher-level components, we used an Intel compute stick, which acts as a decision-maker for the robot. The compute stick runs with a robot operating system (ROS) under an ubuntu environment. For navigation and localization purposes, we used an RPLidar A3, which was mounted on top of block 2. The Arduino at block 2, which acted as an MCU to communicate with each motor controller (Roboclaw) located at the corresponding block. The control signal acted as ROS topics sent from the compstick with the ROS master installed to Arduino to generate the appropriate PWM to robot motors. We used a USB hub that took the Arduino and Lidar cable as inputs and connected them to the Intel compute stick. With sensor information from Arduino and Lidar, the compute stick’s decision passed the navigation commands to the Arduino.

For stable localization, we fused the Lidar’s range information and the IMU data in the robot localization package of ROS. Using the robot’s global position, the proposed navigation algorithm will generate the appropriate path to achieve maximum area coverage. This global path will be passed to the ROS navigation stack wherein the local path planner generates the command velocity for the robot that passes to the local controller (Arduino). The local controller later passes the PWM values to the motor.

### 2.2. Description of hTetran in the Polyabolo-Based Worspace

**Figure 4:**
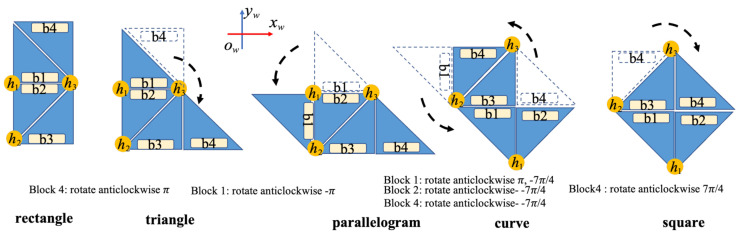
Representation the shapeshifting of hTetran in the workspace.

The prefabricated workspace is divided into a predefined size polyabolo-based connected network in which each cell’s size is equivalent to robotic cubes. The robot inside this workspace is described as a reference 4D coordinate $W(x,y,T,\varphi_{h})$ that includes the gravity center of hTetran platform x,y, the tile *T*, the orientation heading $\varphi_{h}$. The modules and block actions of the hTetran structure with the robot structures of five accessible forms of the four-block areas on the hTetran header inside the workspace are depicted in Figure 4. The required actions of hTetran shapeshifting in the sequence among the available rectangle, triangle, parallelogram, curve, and square around the dynamic axis ID joins presented as $h_{1},h_{2},h_{3}$ is fine-tuned by the necessary point revolutions of the robot block. The hTetran area of a square *b* is denoted by $\left\{x_{b}^{w},y_{b}^{w},\varphi_{b}^{w}\right\}$, where *b* is in the four modules of hTetran $\left(b\in \left\{B_{1},B_{2},B_{3},B_{4}\right\}\right)$ which can be obtained from the robot morphologies inside the workspace. The masses of all modules are distributed among $m_{1},m_{2},m_{3},m_{4}$.

The robot’s related operations, including change shape, linear movement, and heading adjustment, can be systematically described to move the robot between any given waypoints within the working environment. In particular, the aim of the robot’s trajectory to access all reference points is divided into the set of sequenced arrangements of the two reference points. To handle all the reference points *n*, the course pair is described as $p(W_{k}^{s},W_{k}^{g})$, where *k* denotes the considered pair and *s* is the source reference and *g* is the goad reference of the pair *k*. The starting waypoint would have k=1, and the final reference would have k=n−1. Considering the example workspace that includes *n* desired points, n−1 is the number of pairs, and the possible trajectory which connects all the pairs is Ω = n(n−1))/2.

## 3. Complete Path Planning by hTetran the Polyabolo-Based Tiling Platform

### 3.1. Tiling Theory for Polyabolo-Based hTetran

**Figure 5:**
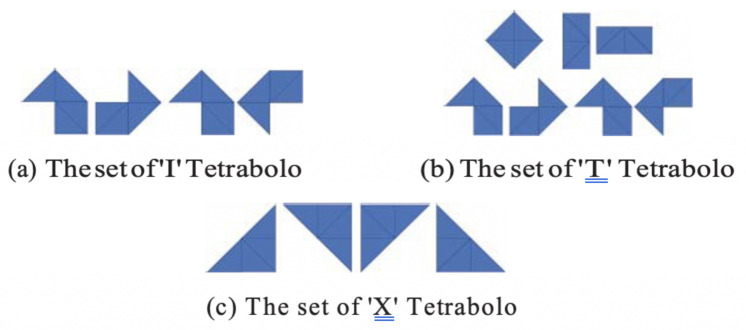
Hinged link between each right triangle for the hinged combination.

The hTetran platform applies the Polyabolo tiling-based path planning during the area coverage of the predefined workspace. The presented work is our initial attempt to implement Polyabolo tiling as a coverage path planning technique in a reconfigurable robot. Specifically, we evaluated the tiling theorems, where they tiled a regular polygonal area using only ‘I’, ‘T’, and ‘X’ tileset pieces. Figure 5 shows the tile pieces that belong to each mentioned tileset. In the experiments, we only used the ‘I’, ‘T’, and ‘X’ tilesets to perform the area coverage. The three theorems that will be used in our experiments are detailed below.

**Theorem** **1.** *A triangle with a base can be tiled with the ‘X’ Tetrabolo only if the number of the triangle either is 2 or is divisible by 2.*

**Theorem** **2.** *A square whose sides have a divisible triangle by 2 and 8, then the square can be tiled using ‘T’ tetrabolo.*

**Theorem** **3.** *An octagonal space with a side that consists of triangles, which gives nonrational values when it is divided by 2, which can be tiled using the ‘I’ tetrabolo.*

### 3.2. Optimal Complete Overage Framework

**Figure 6:**
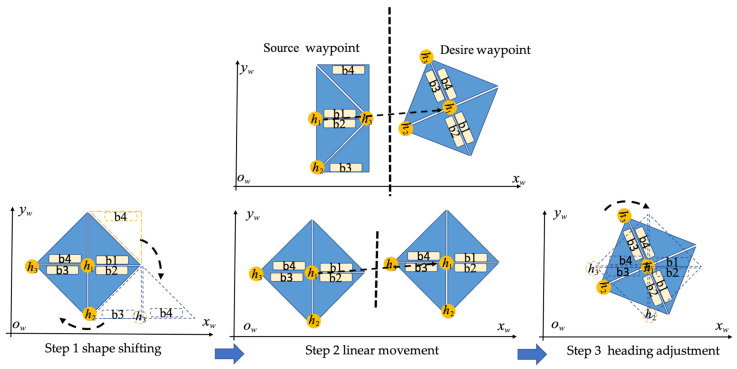
Sequence of 3 actions of hTetran from source $W_{k}^{s}$ with a rectangle shape to destination $W_{k}^{d}$ with a square shape.

The complete coverage framework for the hTetran robot consists of three stages: workspace forming; stage placement; and execution. To explore the set of grid cells after describing the workspace shapes, the backtracking technique [32] approach was applied. Specifically considering a polyabolo-based predefined workspace, and selected morphologies among five available hTetran shapes are arbitrarily set. In the case the searching algorithm cannot sort the accompanying tiles, different perspectives of the previous tile are tried. The same cycle is executed until the four blocks-based tilesets completely cover all the workspace’s free polyabolo-based grid cells. The center of gravity of block 2 of each tile of the tileset is defined at the waypoint to be visited by hTetran. To complete the route connecting all the waypoint inside the workspace, the hTetran derives the optimal trajectory then stores the sequence in the navigation database, as shown in Figure 6. To clear every waypoint’s pair during navigation, hTetran performs three operations, including shapeshifting to the desired shape at the target point; performing a linear movement of the source reference point $W^{s}$ reference target point $W^{d}$; and make the correct orientation of robot heading between the robot’s current direction and the wanted direction at the target. For the presentation of each activity, the required rotations $\theta_{k}$ of each hTetran block to shift between five available shapes are presented in Table 1. The module length of each block in meters during the shapeshifting could be $l_{m}=\sum (l_{1}$ + $l_{2})$ in which $l_{1}$ is equal to length from hinge to the center of mass (COM) of the block during the first rotation and $l_{2}$ is equal to the length from the hinge to the COM of the block during the second rotation. These qualities are shown in Table 2. The required directional adjustment of the hTetran orientation adjustment is characterized by the different heading between the hTetran header at the target reference point $\varphi_{h}^{g}$ and the source reference point $\varphi_{h}^{s}$. From the tables, the required actions linking to the energy usage to shift the robot shape from one specific shape to the desired shape are considerably different. Hence, the optimal shapeshifting order is needed during locomotion.

## 4. Reinforcement Learning Approach for TSP-Based Coverage Path Planning

### 4.1. Energy Aware RL Reward Function

The succession of required operations, including morphology shifting, linear movement, and orientation adjustment during the clearance of a pair of waypoints found by tiling and backtracking techniques, is shown in Figure 6. These operations’ energy usage is estimated by accumulating the actuator’s rotation distance and the individual robot module’s weight. The required energies for linear translation, shape transformation and direction modification are described in Equations (1)–(3). The total robot’s energy spent can then be determined by using the idea of transferring the stage mass from the source reference point $W_{k}^{s}\left(x,y,T,\varphi_{h}\right)$ to the target reference point $W_{k}^{g}\left(x,y,T,\varphi_{h}\right)$, described by the sum of all partial energies as shown in Equation (4):

(1) $$E_{tranl}\left(W_{k}^{s},W_{k}^{g}\right)=\sum_{b=B1}^{B4}m_{b}\sqrt{{\left(x_{b}^{g}-x_{b}^{s}\right)}^{2}+{\left(y_{b}^{g}-y_{b}^{s}\right)}^{2}}$$

(2) $$E_{tranf}\left(W_{k}^{s},W_{k}^{g}\right)=\sum_{b=B1}^{B4}m_{b}\theta_{b}l_{m}$$

(3) $$E_{ori}\left(W_{k}^{s},W_{k}^{g}\right)=\sum_{b=B1}^{B4}m_{b}\left|\varphi_{h}^{g}-\varphi_{h}^{s}\right|l_{m}$$

(4) $$E\left(W_{k}^{s},W_{k}^{g}\right)=E_{tranl}\left(W_{k}^{s},W_{k}^{g}\right)+E_{tranf}\left(W_{k}^{s},W_{k}^{g}\right)+E_{ori}\left(W_{k}^{s},W_{k}^{g}\right)$$

Based on the unique operation of a robot defined as energy functions, the proposed complete path planning is modeled as cleaning the set of predefined waypoint sequences with the target capacity to limit the overall energy usage. The defining problem is the classic TSP, the nondeterministic polynomial time hardness problem. To deal with this NP-hard TSP with many reference points, an indeterminate methodology is presented to infer the Pareto-optima arrangement. This paper deals with the hTetran tile sorting sequence to clear the predefined waypoints using RL and deep recurrent neural networks. With the defined 4D location of the reference points generated by the tiled hypothesis, as the observation space of a finite Markov decision process, we observe the one direction trajectory π, connecting all the reference points (in addition to the original reference points) that have minimal energy usage. A permutation π as the cost of the trajectory is presented as follows:

(5) $$L\left(\pi |O\right)=E\left(W_{n}^{s},W_{1}^{g}\right)+\sum_{k=1}^{n-1}E\left(W_{k}^{s},W_{k}^{g}\right),$$

where the observation space tileset contains *n* reference points $O={\left\{W_{k}\right\}}_{k=1}^{n}$ and each $W_{k}$ store shape and pose of the robot in the predefined workspace. Then, we defined the negative of the trajectory cost described by Equation (5) as the cumulative expected reward r(π|O), which we aim to maximize:

(6) $$\begin{array}{cc} r(\pi |O) & =-L(\pi |O)\\ \; & =-E\left(W_{n}^{s},W_{1}^{g}\right)-\sum_{k=1}^{n-1}E\left(W_{k}^{s},W_{k}^{g}\right) \end{array}$$

### 4.2. Optimization with Reinforcement Learning

We applied the well-known RL-based TSP framework of [33] with the proposed cost functions connecting pairs of 4-dimensional waypoints (x,y,shape,heading) based on the robot kinematic design and operation within the polyabolo tileset generated by tiling theory. Note that the original paper’s cost function uses the 2D Euclidean between two locations inside the workspace. Specifically, we employed the actor–critic methods [34] to learn approximations to both the policy and value functions of the RL problem. Two neural networks were utilized to represent the actor and critic networks, similarly to the work of [30]. Both networks employed the pointer network architecture [35], consisting of a pair of RNNs (encoders and decoders), each containing long short-term memory (LSTM) layers [36] to parameterize the trained policy and value model. For further details on the neural network architecture, we refer to the works of [30,35].

We learn the policy parameters θ of the actor network concerning the training objective, i.e., the expected reference points trajectory given an input observation space tileset as Equation (7):

(7) $$J\left(\theta |O\right)=E_{\pi ∼p_{\theta }\left(.|O\right)}r\left(\pi |O\right).$$

The methods that follow this general schema of learning the policy parameter θ based on the gradient of J(θ|O) with respect to the policy parameter θ are called policy gradient methods, whether or not they also learn an approximate value function [37]. Since we followed the actor–critic methods described in the previous section, a critic network was also utilized to learn approximations to the value function.

In this work, proximal policy optimization (PPO) algorithm [38] was adopted to optimize the policy of the actor pointer network parameters. PPO is the latest modern policy gradient method in reinforcement learning, which is extremely powerful and can be implemented and tuned very simply. Hence, the policy gradient-based objective as Equation (8) is expressed using the PPO’s clipped surrogate function, which offers robust updates throughout the scheme of optimization:

(8) $$\nabla_{\theta }J^{CLIP}\left(\theta |O\right)={\hat{E}}_{\pi ∼p_{\theta }\left(.|O\right)}\left[min\left({\hat{A}}_{t}\nabla_{\theta }r_{t}\left(\theta \right),\right.\right.\left.\left.{\hat{A}}_{t}\nabla_{\theta }clip\left(r_{t}\left(\theta \right),1-\epsilon ,1+\epsilon \right)\right)\right]$$

where the expected value ${\hat{E}}_{t}\left[\cdots \right]$ is the empirical mean across a finite batch of samples, $r_{t}\left(\theta \right)=\frac{\pi_{\theta }\left(a_{t}|o_{t}\right)}{\pi_{\theta_{old}}\left(a_{t}|o_{t}\right)}$ is the probability ratio of the new $\pi_{\theta }$ and the old $\pi_{\theta_{old}}$ policies, ${\hat{A}}_{t}=r\left(\pi |O\right)-b\left(O\right)$ denotes the advantage function, where b(O) represents the baseline, which is used to estimate the expected value of the trajectory cost, thereby reducing the variance of the gradients. If the probability ratio between the new policy and the old policy falls outside the range (1−ϵ)–(1+ϵ), the advantage function will be clipped.

The baseline b(O) we proposed utilizes the same pointer architecture without the final softmax layer, called a critic network. The critic network is parameterized by $\theta_{v}$, where the expected value of the reference points trajectory or the baseline is estimated by the input observation space tileset. This work optimizes the critic network using the stochastic gradient descent of the mean squared error objective between its estimations b(O) and actual reward value of the reference points trajectory r(π|O), which we collect from the most recent episode:

(9) $$J\left(\theta_{v}\right)=\frac{1}{b}\sum_{i=1}^{b}{\left(b\left(O_{k}\right)-r\left(\pi_{k}|O_{k}\right)\right)}^{2}$$

## 5. Experimental Results

### 5.1. RL Training and Trajectory Generation Results

**Figure 7:**
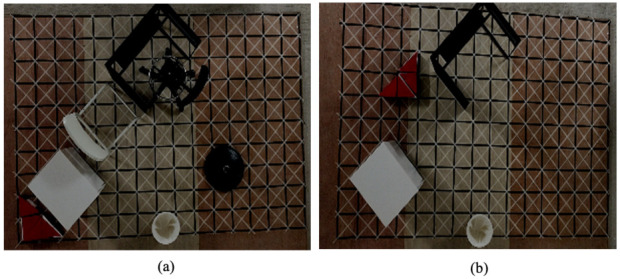
Simulation experimental setup with different layouts: (**a**) scenario 1 environment; and (**b**) scenario 2 environment.

We experimented with the generated directions determined by CPP techniques in reconstructed workspaces with an arrangement of polyabolo tiles. The grid cell was set to the exact shape of an hTetran block as shown in Figure 7. The four linked polyabolo blocks were placed by backtracking technique to represent the reconfigurable robot morphologies inside a specific workspace with arranged obstacles. The obstacle regions were arbitrarily placed and have a value of −1. To show the movement of the hTetran shape, the complicated workspaces that complied with tiling theory were created to fit the robot shape properly. The workspace was designed so that one shape, such as the rectangle or square shape, was impossible to cover completely, but all the hTetran available shapes were exploited to cover the given workspace. Tilesets were created by the arbitrary arrangement of robot shapes inside the predefined workspace by backtracking [32]. Ideal directions were represented as a derived path connecting the tiles with the optimal navigation strategy in terms of energy saving.

We implemented the proposed RL approach using the Tensorflow framework with the pointer network architecture for TSP and changed the policy optimization to PPO loss. All analyses ran on a workstation with the specifications: Intel Center i7-9750H processor and 16 GB Memory with Nvidia Quadro P620 GPU. We tried different parameter sets with 1000 charts of 20, 50, and 100 examples of TSP 4D reference points. The mini-batch was set to 256 arrangements with lengths of 10, 20, and 50. We utilized the proposed energy reward function as described in Equation (6). The α=0.3 was chosen depending on the preliminary trial results. The Adam optimizer [39] with an initial learning rate of 1 × 10${}^{-3}$ was applied to enable the minimization of the cross-entropy loss per batch.

**Figure 8:**
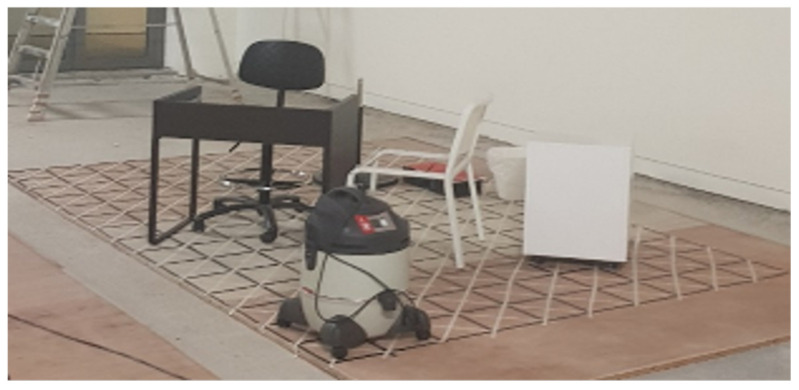
Real environment setup with the worlksape similarly to Figure 9.

**Figure 9:**
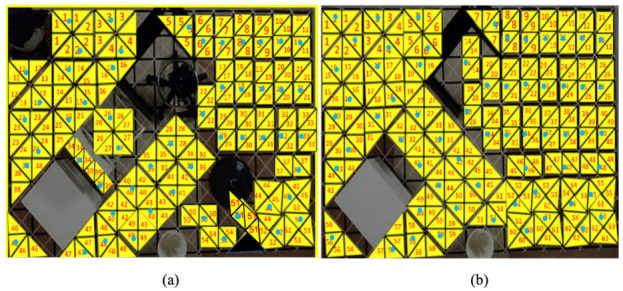
Worksapces tileset arrangements: (**a**) scenario 1 environment; and (**b**) scenario 2 eviroment.

**Figure 10:**
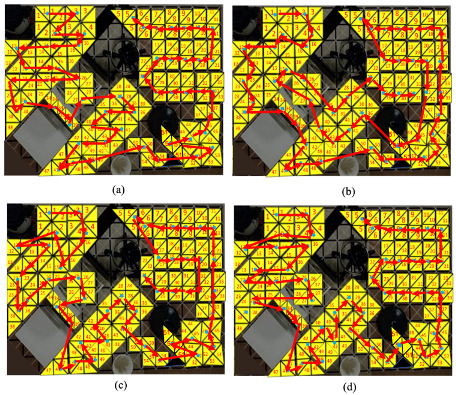
Optimal trajectories generated by tested methods for scenario 1: (**a**) Zigzag; (**b**) Spiral; (**c**) ACO; and (**d**) reinforcement learning-based travel sales problem (RL-TSP).

**Figure 11:**
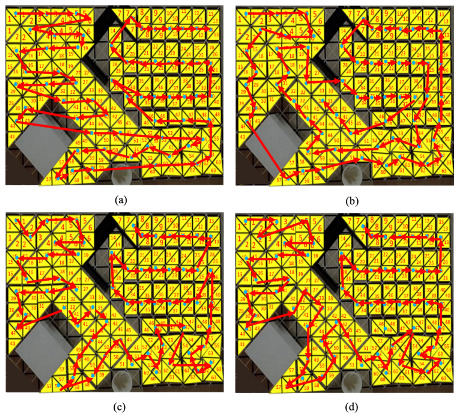
Optimal trajectories generated by tested methods for scenario 2: (**a**) Zigzag; (**b**) Spiral; (**c**) ACO; (**d**) Proposed RL-TSP-based method.

The conventional TSP techniques consisting of zigzag, spiral, and evolutionary-based ant colony optimization were compared with the RL-based proposal technique. A description of the total number of waypoints, including the testbed workspace, is shown in Figure 8. Figure 9 presents the productivity-driven awareness of the RL-based strategy for different workspaces and tile arrangements. Figure 10 and Figure 11 show the correlation direction of all the techniques tried for the obstacle workspace tilesets of Figure 9a,b, respectively. Table 3 represents the values for the cost and time comparison. The cost function in Equation (6) is used in both RL and evolutionary technique ACO [40]. One constraint condition has been added to the zigzag- and spiral method while selecting the connected pair of waypoints, which is that if the Euclidean distance between two waypoints should be less than the defined threshold of 5. We excluded the waypoint 56 of Figure 9 and waypoint 46 in Figure 10 during the path planning since they are in the corner of the workspace which can not be accessed by the robot.

From the information in Table 3, all the evaluated strategies have practically identical Euclidean lengths. Similar to [33] for the TSP with the small number of waypoints, the arrangement of the RL-TSP system reaches the ideal cost level simulated workspaces. The improvement between RL-TSP and ACO fluctuates slightly with a relatively small reference waypoints workspace. Despite completing the fastest time, zigzag and spiral processes using the basic crosswise pairs connecting by linear lines in the left and right order, cost weight results are slightly higher than the evolutionary-based techniques. The runtime and cost weight of these path-searching techniques are higher than those of the GA and ACO systems. The RL-based method gradually focuses on the mathematical estimates of cost weight and execution time. The RL-based technique’s cost weight was around 7% better than ACO, the second-best optimal method.

Considering the strategy trajectories generated by the RL-based method, two hTetran shapes with equal morphologies and less directional orientation differences were chosen to connect in priority order inside the detected trajectory as shown in Figure 10d and Figure 11d. With the advancement of the comparative cell title in a timely manner, the RL sometimes gives a higher need to select the reference point that comes with the shape with lesser directional changes. For example, with shapes equivalent to the rectangle of tile 34 in Figure 9a, RL-CPP links tile 39 and not tile 27. Moreover, from tile 25 of the triangle shape, it selects tile 26 that is the same triangle shape, even though tiles 24,34,38 of square shapes have the shortest Euclidean distance to become the following tile because there is no need to transform the shape and correct the heading of the robot.

Furthermore, the RL-CPP selects the next tile of the directional trajectory, considering the fewer blocks to transform the robot shape to the desired morphology. For instance, from tile 54 of rectangle shape, RL links to tile 51 of the parallelogram that requires the module $B_{1}$, and $B_{4}$ is turned around the axis $h_{1}$, $h_{3}$ with revolutions of −pi, pi rad and magnitudes of $l_{1}$ and $l_{1}$, respectively, instead of tile 52 with the curve shape, which requires the three modules $B_{1}$, B2, and B3 to rotate the revolutions of −π/2,−7π/4,−7π/4 rad and magnitudes of $l_{1},l_{1},l_{1}$ around the axes $h_{1},h_{2},h_{3}$, respectively. Due to the reduced steps of transforming the robot shape into the desired reference point, the proposed CPP technique is able to find the best reward strategy during the optimization.

### 5.2. Real Environment Testbed

During the real workspace trials with the paths that were generated, the robot’s energy and time to clear the waypoints found in the sorted data set were evaluated. The robot was placed in continuous self-government and exploration mode to adjust its COM for each of its characteristic reference points, reinforcing its ideal area and shape. The robot roadmap works with the node components and topics provided in the ROS framework. The development requirement robot locomotion of the adaptive feedback control (PID) was developed in our previous work for tiling robot [41]. Once the title was identified, the motor controllers drove servo motors to the direction so that the locomotion units of four blocks were aligned with the direction of the waypoints, then activate DC motors to generate the linear motions.

The fused localization from the different laser-based odometry sensors, IMU, and wheel encoder-based odometry by the Kalman EKF method which enhances the robot’s understanding of the current area even if any sensors are against hardware failure or noise interference. The robot maintains a safe distance from the obstacles throughout the route. The tiles number 34 and 39 in the workspace as Figure 9a represent the robot’s ability to overcome the narrow space. The hTetran’s energy usage was derived using current sensors that communicate with the robot’s battery power 14.4 V, 1000 mAh. The current sensor was fixed at the rate of 10 kHz. The maximum speed of DC motors was regulated to 50 rpm.

Numerical comparisons of energy and time usages of the aforementioned techniques are shown in Table 4. From the given values, one can observe that if the hTetran implements the trajectory as demonstrated, with a lower cost weight, less energy and time are consumed. The method that archived the optimal energy and time usage was the recommended RL-based method. This strategy’s profitability is about 7%, better than ACO as the second-best method. The outcomes show that the proposed path planning method is a plausible process that could be implemented in order to spare the energy spent, specifically for the hTetran robot.

Energies for a single operation between shifting, correcting direction, and linear movement to complete the testbed area are provided in Table 4. According to the results, straight movement consumes the most battery capacity since all three DC motorscarrying the whole robot’s weight, and all guide servo motors holding the robot blocks are activated during linear movement. Shapeshifting is the second place of energy usage; in addition, the robot heading offset adjustment represents a third of the battery usage.

## 6. Conclusions

The reconfigurable tiling hTetran delivers a viable solution to cover different predefined workspaces by saving both energy and time by about 7%, better than the state-of-the-art CPP methods. RL-based CPP was systematically evaluated to infer the most restrictive direction for the proposed TSP than the conventional-based strategies. The proposed CPP is suitable for being skillfully applied to other tiling platforms such as diamond, hexagon, and rhombus shapes. This paper’s proposed CPP framework is the initial step to realizing the feasible RL-based TSP framework into the cleaning business, where the fixed-structure platforms present the limitations in the area coverage of complex workspaces.

Since the robot is in the developing stage of the operation within relatively small workspaces, strategic testing in larger workspaces to confirm the proposed RL-TSP CPP framework needs to be further analyzed. On the other hand, the other tiling robot with a different shape such as diamond, rhombus, the hexagon can be combined to form a flexible reconfigurable platform to troubleshoot instructions to specific sub-maps. The investigation opens up various potential discoveries, including ideal control techniques, mechanics, and system designs. The future works could be as follows:

- A model for assessing necessity in a dynamic workspace;
- The autonomous tuning for hyperparameters of RL frameworks;
- Multi-target RL;
- Increased autonomy of considerable distance with the robot stage tiled movement;
- Consideration of robot locomotion and environment friction.

## Figures and Tables

**Table 1:** Required turning angle $\theta_{k}$ of hTetran modules during shapeshifting.

	*W^d^*	Rectangle $B_{1}B_{2}B_{3}B_{4}$	Triangle $B_{1}B_{2}B_{3}B_{4}$	Parallelogram $B_{1}B_{2}B_{3}B_{4}$	Curve $B_{1}B_{2}B_{3}B_{4}$	Square $B_{1}B_{2}B_{3}B_{4}$
*W^s^*						
**Rectangle**		0 0 0 0	0 0 0 π	−π 0 0 π	−π/2 −7π/4 −7π/4 0	0 0 −7π/4 (π, −7π/4)
**Triangle**		0 0 0 −π	0 0 0 0	0 0 0 −π	−7π/4 −7π/4 0 0	−7π/4 −7π/4 0 −7π/4
**Parallelogram**		π 0 0 −π	0 0 0 π	0 0 0 0	(π, −7π/4) −7π/4 0 −7π/4	(π, −7π/4) −7π/4 0 0
**Curve**		π/27π/4 7π/4 0	7π/47π/4 0 0	(−π, 7π/4) 7π/4 0 7π/4	0 0 0 0	0 0 0 −7π/4
**Square**		0 0 7π/4 (−π, 7π/4)	7π/47π/4 0 7π/4	(−π, 7π/4) 7π/4 0 0	0 0 0 7π/4	0 0 0 0

**Table 2:** Turning length of the hTetran modules when shapeshifting.

	*W^d^*	Rectangle $B_{1}B_{2}B_{3}B_{4}$	Triangle $B_{1}B_{2}B_{3}B_{4}$	Parallelogram $B_{1}B_{2}B_{3}B_{4}$	Curve $B_{1}B_{2}B_{3}B_{4}$	Square $B_{1}B_{2}B_{3}B_{4}$
*W^s^*						
**Rectangle**		0 0 0 0	0 0 0 $l_{1}$	$l_{1}$ 0 0 $l_{1}$	$l_{2}$$l_{1}$ 0 $l_{1}$	0 0 $l_{1}$ ($l_{1}$, $l_{2}$)
**Triangle**		0 0 0 $l_{1}$	0 0 0 0	$l_{1}$ 0 0 0	$l_{2}$$l_{1}$ 0 0	$l_{2}$$l_{1}$ 0 $l_{1}$
**Parallelogram**		$l_{1}$ 0 0 $l_{1}$	$l_{1}$ 0 0 0	0 0 0 0	($l_{1}$, $l_{2}$) $l_{1}$ 0 $l_{1}$	($l_{1}$, $l_{2}$) $l_{1}$ 0 0
**Curve**		$l_{1}$$l_{1}$$l_{1}$ 0	$l_{1}$$l_{1}$ 0 0	($l_{1}$, $l_{2}$) $l_{1}$ 0 $l_{1}$	0 0 0 0	0 0 0 $l_{1}$
**Square**		0 0 $l_{1}$ ($l_{1}$, $l_{2}$)	$l_{1}$$l_{1}$ 0 $l_{1}$	($l_{1}$, $l_{2}$) $l_{1}$ 0 0	0 0 0 $l_{1}$	0 0 0 0

**Table 3:** Cost weight and running time of generating trajectories for simulation workspaces.

Approach	2D Distance (m)	Total Cost Weight (Nm)	Running Time (second)
Zigzag	51.43	382.26	0.05
Spiral	50.91	384.32	0.06
ACO	49.42	322.15	6.21
RL	49.09	315.36	2.16

**Table 4:** Energy and time usages in real testbed workspace.

Method	Costweight	Summation	Translation	Transformation	Orientation	Travel
-	(Nm)	Energy (J)	Energy (J)	Energy (J)	Energy (J)	Time (second)
Zigzag	382.26	63.26	32.39	19.52	11.35	1683
Spiral	384.32	60.26	30.32	19.11	10.83	1679
ACO	322.15	53.59	25.51	17.95	10.13	1244
RL	315.36	51.15	26.24	15.56	9.35	1212

## Data Availability

Not applicable.

## Abbreviations

The following abbreviations are used in this manuscript:

ACO	Ant Colony Optimization
CPP	Coverage Path Planning
GA	Genetic Algorithm
GRU	Gated Recurrent Unit
LSTM	Long Short-Term Memory
PPO	Proximal Policy Optimization
RL	Reinforcement Learning
RNN	Recurrent Neural Network
ROS	Robot Operating System
TSP	Travelling Salesman Problem
